# Positive Results Using Variable Fixation in Medial Opening Wedge High Tibial Osteotomies in Patients with Unilateral Knee Osteoarthritis: An Observational Clinical Investigation

**DOI:** 10.3390/jcm13247707

**Published:** 2024-12-17

**Authors:** Christian Colcuc, Thomas Vordemvenne, Georg Beyer, Philipp Leimkühler, Dirk Wähnert

**Affiliations:** Bielefeld University, Medical School and University Medical Center OWL, Protestant Hospital of the Bethel Foundation, Department of Trauma Surgery and Orthopedics, 33617 Bielefeld, Germany; thomas.vordemvenne@evkb.de (T.V.); georg.beyer@evkb.de (G.B.); philipp-julian.leimkuehler@evkb.de (P.L.); dirk.waehnert@evkb.de (D.W.)

**Keywords:** osteoarthritis, knee, HTO, high tibial osteotomy, Variable Fixation Locking Screw, VFLS, dynamization, fracture healing

## Abstract

**Background:** Medial opening wedge high tibial osteotomy (HTO) treats medial knee osteoarthritis by realigning the knee joint, though it still carries quite a high risk of complications. A new Variable Fixation Locking Screw technology, designed to gradually reduce construct stiffness and promote bone healing, aims to address these issues. This observational study evaluates the safety and effectiveness of this innovative approach in improving clinical outcomes. **Methods:** Data were prospectively collected on a cohort of the first ten consecutive patients (over 18 years of age) who underwent corrective medial opening wedge high tibial osteotomy using Variable Fixation Locking Screws (VFLSs). The procedure followed the standard surgical technique, with osteotomies stabilized using a Tomofix plate and a combination of standard locking screws and VFLSs. This study aimed to evaluate outcomes such as fracture healing, patient safety, and procedural success at 6 and 12 weeks and at 6 months. **Results:** No complications, side effects, or need for implant removal were observed. By six months, 70% of patients showed radiographic and clinical healing, and 100% of patients achieved full functional recovery without any issues like length discrepancy, instability, pain, or joint stiffness. **Conclusions:** This first clinical observation study indicates that Variable Fixation Locking Screws are safe and effective for medial opening wedge high tibial osteotomies, showing promising results in reducing the risk of delayed closure or non-closure of the wedge. Further studies with a larger patient population are needed to confirm their effectiveness.

## 1. Introduction

Medial opening wedge high tibial osteotomy (MOWHTO) has established itself as a reliable surgical procedure for the treatment of knee joint deformities and disorders, particularly in younger, active patients [1]. This technique is particularly effective for patients with medial knee osteoarthritis characterized by malalignment and provides a viable approach to correcting the limb axis and optimizing load distribution across the joint. Indications for a high tibial osteotomy include malalignment of the knee joint with relevant impairment of the biomechanical function of the knee joint. Malalignment can be caused by a variety of factors, including post-traumatic deformity [2], chronic overuse, aging, rheumatic disease, or congenital malalignment [3]. The primary goal is to realign the knee joint by shifting the weight-bearing axis from the damaged medial compartment to the healthy lateral compartment, thereby relieving pain and slowing the progression of the disease [4]. Technically, it is a medial osteotomy of the proximal tibia that remains incomplete laterally, allowing the lateral cortex to serve as a hinge around which the distal fragment can rotate. A second ascending osteotomy is performed above the tuberosity to allow the first osteotomy to be opened. Its biomechanical impact not only affects tibiofemoral pressures, but alters the tibial slope and changes the position of the patella. These are all critical parameters to control in order to mitigate the risks associated with this procedure [5]. In fact, as little as 1° malalignment in the coronal plane has been found to result in an additional 12% body weight being distributed to the medial compartment [6]. Several studies have reported significant improvements in clinical outcome scores (Knee Injury and Osteoarthritis Outcome Score—KOOS) and visual analog scales for pain (VAS) reported by patients after the procedure during the clinical course [4]. Identified key factors influencing outcomes are careful patient selection (patient age, degree of osteoarthritis), accurate planning and surgical execution, and rigorous postoperative management [4].

Patients have high expectations for postoperative outcomes following medial opening wedge high tibial osteotomy, particularly in terms of pain reduction, restoring knee functionality, and returning to work [7,8]. Surgeons also have high expectations of HTO, but they differ from patients’ expectations. While pain reduction is the top priority for surgeons and patients, their expected results in terms of delaying/preventing knee arthroplasty and returning to athletic activity are different from patients’ expectations reported in the literature [9]. For instance, surgeons expected their patients to return to their previous levels of exercise with restrictions or to lower levels of exercise, while more patients expected to return to their previous levels of exercise without restrictions [8,9]. This should be considered in the preoperative informed consent process [9]. Sohn et al. examined factors predicting patient dissatisfaction after MOWHTO and found that a Kellgren and Lawrence score of level 4 was significantly associated with dissatisfaction [10]. When comparing Unicompartmental Knee Replacement (UKR) versus HTO, a systematic review on 38 clinical studies [11] has shown that UKR produced less postoperative pain and a superior Western Ontario and McMaster Universities Osteoarthritis Index (WOMAC) score, whereas HTO offered an extended range of motion and lower revision rate but a higher complications rate. A systematic review by Miltenberg et al. [6] on 71 studies comprising 7836 patients highlighted an overall intraoperative HTO complication rate of 5.5%, with lateral hinge fractures being the most common complication during medially based HTOs (9.1%). Postoperative HTO complications were observed in about 6.9% of cases, with superficial infections being the most common (2.2%), followed by non-unions or delayed unions, occurring in about 1.9% of cases [6], and hardware-related complications such as plate or screw loosening or breakage in approximately 10% of patients [6].

The rigid locking construct used to stabilize the medial opening wedge high tibial osteotomy is often considered a factor that may delay bony consolidation. This can lead to pain and delayed remobilization of the patient. The degree of rigidity of bone segment stabilization has a significant impact on variables, substantially affecting the course of healing such as fracture gap strain [12,13,14,15]. The usage of new locking screw concepts that provide some degree of additional interfragmentary mobility has already been proposed as a method to promote healing and has been tested, for example, on the distal tibia [16,17]. Building on this understanding, a novel osteosynthesis concept, termed Variable Fixation, was developed [18]. In Variable Fixation, the stability provided, for example, by the plate-and-screw construct is not constant but, starting from the conditions imposed by the surgeon during implantation, features a naturally evolving and controlled dynamization over time. Its development was based on three opportunities for improvement grounded in fundamental mechanobiological principles [18]. First, growing evidence in the literature demonstrates that different phases of fracture healing profit from different levels of stability. Enhanced stability during the inflammatory phase facilitates angiogenesis and promotes the differentiation of cells into an osteogenic lineage. In contrast, controlled dynamization during matrix deposition stimulates the formation of fibrocartilage and bone and finally the differential increase in mechanical stimulation shall be limited in order to allow calcification and remodeling of the newly deposited tissue. Second, the pursuit of a single definitive value for gap strain to guide fracture healing has long been a focus in the research field. However, such a universal value simply does not exist. The strain experienced by each cell depends on a combination of factors, including the local fracture gap size, fixation stiffness, applied load, and the distance from the implant. Applying a uniform strain to all cells in the gap with a specific (yet unknown) strain value has proven impractical. Instead, transitioning from rigid fixation to a controlled degree of dynamization, while simultaneously altering the trajectories of the bone segments, can gradually increase the strain across all cells within the gap tissue. Third, adjacent to the fracture site, healthy, vascularized bone contains all the necessary tissues, cells, and signaling mechanisms to drive and complete the healing process. These bone segments serve as an optimal pre-existing scaffold and the opportunity lies in effectively activating and leveraging this inherent regenerative capacity to support the closing of the fracture gap. Biomechanical and preclinical investigations provided encouraging results [19,20] but evidence on the safety of their usage on humans is still missing and shall be produced to allow for larger studies focused on the efficacy of this new locking screw technology. The purpose of the present observational study was thus to report on the safety of this new dynamization concept in medial opening wedge high tibial osteotomy and to evaluate the clinical results at predefined time points.

## 2. Materials and Methods

**Figure 1:**
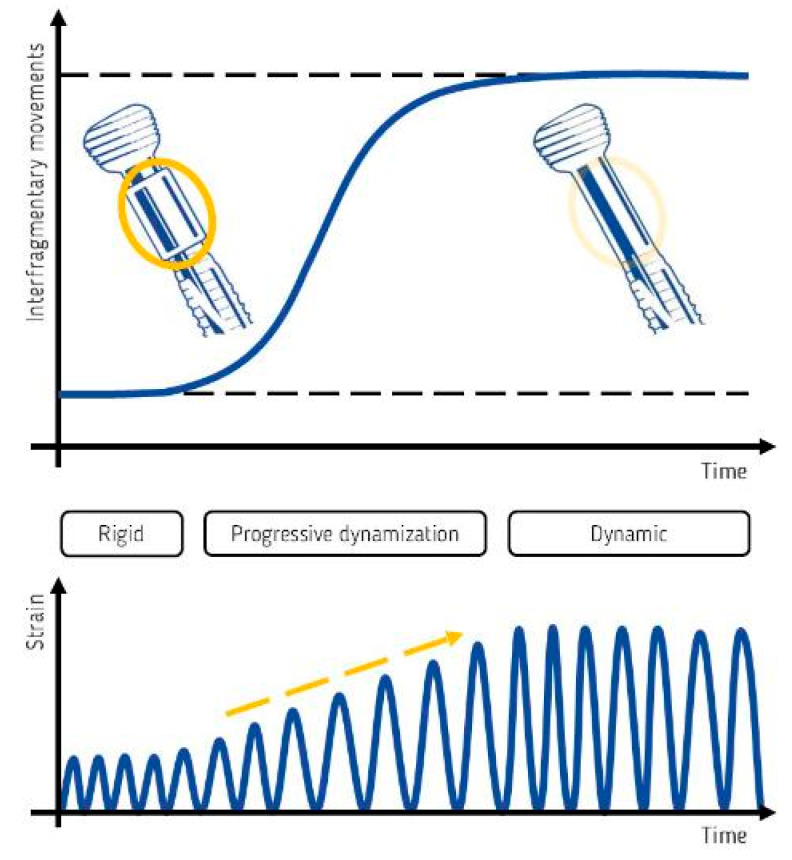
A conceptual schematic showing how the support provided to the cis cortex by the Variable Fixation Locking Screw evolves over time, along with its impact on interfragmentary movements (top graph) and strain (bottom graph). Initially, a stable and well-centered resorbable sleeve (yellow circle) minimizes both interfragmentary movement and strain. As the sleeve gradually degrades, the support from the VFLS progressively decreases, resulting in a controlled increase in interfragmentary displacement and strain (yellow arrow). This gradual change is limited by design, allowing the newly formed bone to complete its calcification and remodeling processes [14,18,19,20].

Data have been prospectively collected on a cohort made of the first consecutive ten patients treated for corrective limb alignment using Variable Fixation Locking Screws (VFLS^®^, Biomech Innovations AG, Nidau, Switzerland) in medial opening wedge high tibial osteotomies. Variable Fixation Locking Screws feature an advanced resorbable sleeve that is implanted in the cis cortex, and initially provides stability comparable to that achievable using standard locking screws [19]. The sleeve gradual resorption leads to a decreased support of the cis cortex and thus to a gradual reduction in the stiffness of the entire construct [19] and to increased mechanical stimulation of bone healing [20] (Figure 1).

All patients recruited for this observational investigation have been treated between October 2022 and August 2023 by the same surgeon (C.C.) at our hospital. Inclusion criteria for this study were as follows: corrective osteotomies with the ability to use 5.0 mm screws in the cortical bone, age of 18 years or older at the time of surgery, ability to walk independently prior to surgery, and willingness and ability to follow the postoperative protocol and return for follow-up. Important exclusion criteria for this study were the following: severe systemic disease, chronic inflammatory disease, active malignancy, osteitis or osteomyelitis, allergic reaction to Ti alloy, lactic, or glycolic acid, lack of bone substance or poor bone quality that, in the opinion of the surgeon, makes locked plate fixation impossible, and the addition of a bone graft, bone substitute, or bone morphogenetic proteins (BMPs). The complete eligibility criteria are reported in (Supplementary Table S1). After having signed the informed consent, patients underwent medial opening wedge high tibial osteotomy according to the standard surgical technique. For preoperative planning, all patients received anteroposterior and lateral radiographs of the affected knee, as well as an axial patella view. These views were used to assess the current status of the knee joint as well as the degree of osteoarthritis according to the Kellgren and Lawrence Score. The extent of the necessary osteotomy was planned preoperatively with mediCAD (mediCAD Hectec GmbH, Altdorf/Landshut, Germany) using a bilateral full-leg scan. Prior to osteotomy, all patients underwent staging arthroscopy of the knee in the same surgery. Under fluoroscopic control, two parallel 2.5 mm Kirschner wires inserted into the lateral cortex were positioned to define the osteotomy plane. These were used to perform the biplanar technique osteotomy according to the preoperative plan using an irrigation-cooled saw blade. The transverse osteotomy ran across the posterior two-thirds of the bone, leaving the ventral third intact. A second, ascending osteotomy in the coronal plane was performed using a thinner saw blade, leaving intact the attachment of the tibial patellar tendon. Anatomical structures dorsal to the posterior bone surface were protected by means of a Hohmann retractor. Osteotomy chisels were inserted into the osteotomy, gradually opening the osteotomy until the planned opening height. The osteotomy gap was not filled with any material, either as an autologous graft or as a bone graft substitute. Osteotomies were then stabilized using a Tomofix plate (Synthes, Solothurn, Switzerland), three standard locking screws (Synthes, Solothurn, Switzerland) in the proximal tibial segment, and three Variable Fixation Locking Screws (VFLS^®^, Biomech Innovations AG, Nidau, Switzerland) in the distal tibial segment. For all patients, the cut-to-suture time has been recorded and the surgeon was asked to rate the handling of the screws on a bad, satisfactory, good, and excellent scale.

Standard anteroposterior and medial–lateral X-ray projections taken at day one after the surgery have been used as the baseline for the follow-up of the fracture healing. In addition, a full-leg scan was performed postoperatively to control for correction, in accordance with our clinical routine. The recommended postoperative management consisted of daily heparin injections as pharmacological thromboembolism prophylaxis until full weight-bearing was achieved. An early weight-bearing protocol with 20 kg partial weight-bearing at 6 weeks postoperation was allowed [21,22]. No support of cartilage cell regeneration, pain management, application of drainages or Tourniquet, and no additional support of bone consolidation were used [23]. The same standard X-ray projections plus a clinical control have been performed at time points 6 and 12 ± 1 weeks and 6 ± 1 months.

Parameters of interest at each time point were as follows: full compliance with aftercare, detection of adverse events affecting patient safety, detection of complications, identification of potentially new unknown side effects on patient health, healing of the fracture visible on radiographs, and clinical fracture healing assessed by the surgeon (C.C.). Procedural success was defined as a return of the bone segment to function without bone segment length discrepancy, instability, pain, or joint stiffness.

The standardized and highly repeatable treatment limited the study size to the first ten consecutive enrolled patients. Nevertheless, the small sample size limits the generalizability of the results of this research, although we have taken all measures to standardize the entire procedure. The qualitative comparison with the literature data is possible because the usage of Variable Fixation Locking Screws is the only variable introduced with respect to standard treatment.

Data were collected on paper-based case report forms in a pseudonymized format, and after all participants had completed the 6-month follow-up the results were digitized onto an Excel spreadsheet for further analysis. Quantitative variables have been analyzed via descriptive statistics while the occurrence of qualitative assessments has been expressed as a percentage of the study population. Microsoft Excel (Microsoft Excel for Mac version 16, Microsoft Cooperation, Redmond, WA, USA) was used for statistical analyses.

## 3. Results

The mean age of the 10 patients (9 male, 1 female) was 47.2 years (SD 6.9 years, median 49 years, IQR 10 years). The wedge opening ranged from 4 to 9 mm, with a mean opening of 7.1 mm and a standard deviation of 1.7 mm (median 7 mm, IQR 3 mm). The mean operative time was 92 min (SD 10 min). The median of the operative time was 90 min (IQR 8 min). The handling during implantation of the new screw has been rated excellent by the surgeon in 100% of the cases.

**Figure 2:**
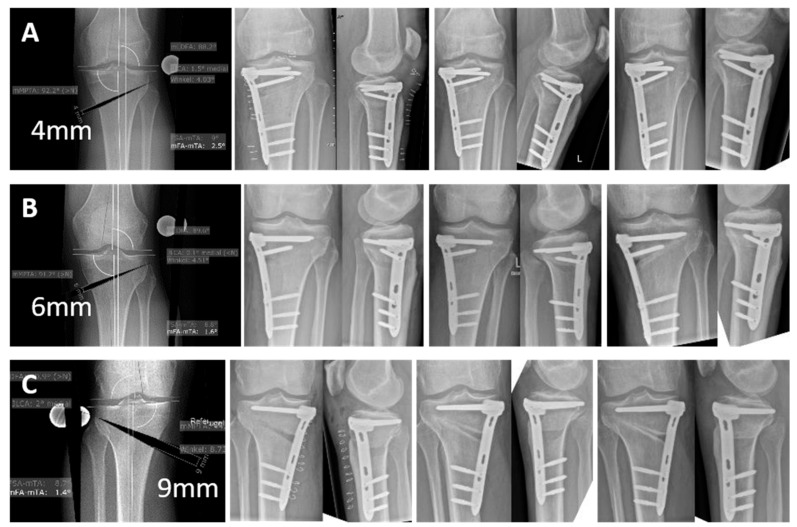
The figure shows 3 exemplary cases of HTOs that were treated with VFLS. The first image in the series shows the pre-operative planning in each case, followed by the X-ray checks post-operatively, after 12 weeks and 6 months. (**A**) 4 mm opening wedge, (**B**) 6 mm opening wedge, (**C**) 9 mm opening wedge.

All patients provided full compliance with aftercare. No adverse event, no complication, and no new unknown side effects on patient health have been detected during this study. The removal of implants due to pain or discomfort was not necessary in any patient up to 6 months postoperatively and up to 12 months in five patients and up to 22 months in one patient that had already reached this follow-up. At 6 weeks post-operation, 10% of patients showed radiographic and clinical fracture healing and one patient showed a hinge non-dislocated type 1 [24] fracture (1/10). At 12 weeks, 30% of patients showed complete and 70% adequate radiographic healing while 60% of patients showed a full return to function without length discrepancy, instability, pain, or joint stiffness. At 6 months, 70% of patients showed complete and 30% adequate radiographic healing while 100% of patients showed a full return to function without length discrepancy, instability, pain, or joint stiffness (Figure 2).

## 4. Discussion

This study presents the first clinical results concerning the use of Variable Fixation Locking Screws. Their application in high tibial osteotomies provides valuable insights into their safety, effectiveness, and biomechanical performance. The findings indicate that the implementation of this novel locking technology is both safe and reliable, with no adverse events, complications, or previously unknown side effects observed throughout the duration of this study. Furthermore, the consistent achievement and maintenance of the planned wedge opening across all patients highlights the capability of these devices to support surgical objectives effectively. The progressive dynamization feature of VFLSs did not interfere with the surgeon’s goal of achieving and maintaining the desired limb alignment, demonstrating the compatibility of this innovative osteosynthesis concept with well-established surgical techniques. An advantage of this technology is its effortless integration into standard surgical workflows, requiring no additional instruments, time investment, or modifications of the surgical technique. The usage of VFLSs also does not require the use of biomaterial wedges [25,26] and the absence of operational delays further support the practical applicability of these screws in clinical settings.

With the increasing life expectancy and more active lifestyles among older adults, the approach to managing knee osteoarthritis (OA) and other degenerative joint conditions has shifted. Medial opening high tibial osteotomy is gaining prominence as a preferred surgical option for this demographic, as it offers several advantages over more traditional treatments such as total knee replacement [27,28]. The complication rate of this technically demanding procedure with overall good clinical results can be decreased, starting in particular from the occurrence of delayed union. The use of bone graft substitutes or void fillers to reduce the risk of delayed union or non-union of the wedge has not resulted in consistent recommendations. Studies comparing similar standard plate-and-screw systems from different manufacturers have demonstrated that there are no significant clinical or radiological differences [29], suggesting the need for the introduction of new concepts.

From a theoretical point of view, the usage of fixation devices stimulating the apposition of bone in the gap might help reach clinical healing earlier and decrease the risk of developing some of the complications typical of HTO. A plausible concern that could arise is that dynamizing an incomplete osteotomy might consistently cause a fracture of the lateral hinge and loss of alignment. In this limited case series, we report that in the only case where a lateral hinge fracture type 1 occurred the fragments kept their relative position under the standard postoperative loading regime and healing occurred with no pain in the expected timeframe.

This observational study presents a unique opportunity to directly compare our preliminary findings with the existing literature, as the sole distinguishing variable between the data reported here and the standard fixation technique is the utilization of VFLSs within the diaphyseal region. Compared to the state-of-the-art healing, the usage of Variable Fixation Locking Screws has a positive effect on the closure of the osteotomy gap. The range of complications associated with fixing medial opening wedge high tibial osteotomies with the same plate and standard locking screws ranges very much based on the criteria used. For example, Niemeyer reported a cumulative rate of major complications of 4.6% [30] while Yauuhci reported a cumulative rate of major complications of up to 24% [31].

**Figure 3:**
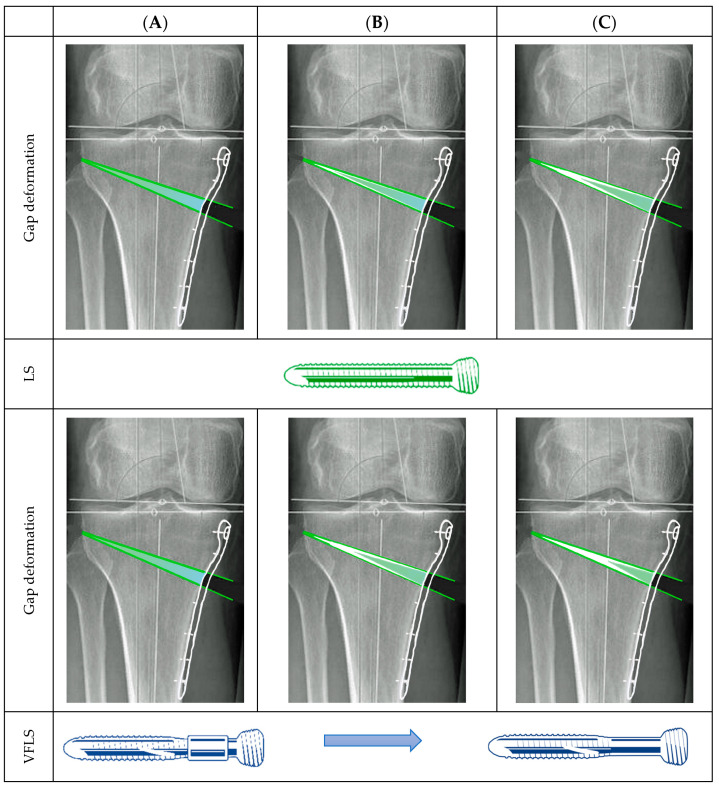
This image illustrates the expected strain distribution within the osteotomy gap. Green areas indicate where the strain is expected to support new bone formation, while light-blue areas show strain levels that are likely too low to promote bone apposition (white). (**A**) Initial strain conditions at the start of treatment using both standard locking screws (LSs) and Variable Fixation Locking Screws (VFLSs). Strain levels with LS fixation decrease over time, potentially leaving parts of the gap unfilled for extended periods. (**B**,**C**) As the degradable sleeve of the VFLS resorbs, gap strain conditions evolve, progressively increasing the likelihood of reaching strain levels conducive to bone formation throughout the entire gap.

The wedge-shaped osteotomy serves as an interesting and robust model for studying the biomechanical effects of progressive dynamization during bone healing (Figure 3). This model provides a unique opportunity to evaluate differential mechanical influences within the same individual across various gap dimensions. Specifically, it allows for the examination of the healing response in a small lateral gap near the hinge, a larger medial gap adjacent to the medial cortex, and the range of intermediate gap dimensions in between. When standard locking screws are employed, weight-bearing forces result in a significant strain on the lateral, extremely small, portion of the osteotomy gap. In contrast, the medial portion, situated under the fixation plate, experiences a progressively diminishing strain during the healing process as the consolidation of the lateral and middle portion progress. This uneven distribution of mechanical strain can lead to delayed or incomplete closure of the medial gap, as the reduced strain stimulus often fails to reach the threshold required for initiating and concluding bone regeneration. In contrast, the use of devices based on the Variable Fixation concept introduces a mechanism of controlled strain increase over time. The initial stability, followed by the gradual decrease in construct rigidity, increases the chances to reach the strain environment, falling within the range that stimulates osteogenic activity along the entire fracture gap. As a result, the likelihood of uniform bone deposition across the entire fracture gap increases, promoting more effective consolidation of the osteotomy.

Overall, the outcomes of this initial case series are positive, as patients’ perceptions of benefits from the surgical procedures have been validated through imaging, pain reduction, and functional improvements and no complications have been detected. By twelve weeks, X-ray evidence of the consolidation process was present in all patients, with nearly 30% showing completion of this process. By six months, 70% of the patients achieved full radiological consolidation and, most importantly, all patients experienced procedural success, with full knee function restored and no length discrepancy, instability, pain, or joint stiffness. In contrast to the relatively high removal rate reported in the literature [32], in our short case history it was not necessary to remove implants for pain relief for up to almost two years after surgery. A 6-month postoperative period is quite short after high tibial osteotomy, but since the 10 cases presented are the first cases worldwide treated with this new stabilization technique, these results are of scientific interest. Patients will be followed-up to obtain medium- and long-term results. In particular, complication rates and revision rates will be of great interest.

Alternative screw concepts have been already developed and introduced to facilitate controlled micromotion at the fracture site, also based on the assumption that mechanical stimuli promote bone healing. Examples of such screw technologies include far cortical locking (FCL) [33], dynamic locking screws (DLS) [34], and over-drilling of the near cortex [35]. Far cortical locking achieves micromotion at the fracture gap by positioning the screw threads to engage only the far cortex, while the dynamic locking screws incorporate mechanical features that, despite engaging both cortices, enable some micromovements. Over-drilling of the near cortex involves using standard locking screws but creating a larger bore in the proximal cortex, allowing the screw to flex and accommodate minor movements. Unlike devices based on the concept of Variable Fixation, which are designed to gradually decrease construct stiffness over time, these screw concepts simply provide an immediate, constant, lower-stiffness fixation of the construct, aiming to strike a general additional interfragmentary displacement thought to promote callus formation. Unfortunately, the relatively promising biomechanical and preclinical results [33,34,36,37,38,39,40] have not been replicated in patients. Achieving precise alignment of screw shafts within the corresponding holes in the cis cortex remains a significant challenge in clinical settings. Even slight deviations during screw placement and construct assembly can result in an uneven load distribution among the screws, creating localized stress concentrations and uneven screw distances with respect to the relative cis hole. Such conditions can lead to the mechanical overload of some screws, ultimately increasing the likelihood of screw failure. Furthermore, the variability in centering precision across different screws and cases contributes to inconsistent and unpredictable clinical results [17,41,42,43,44,45,46,47,48]. In contrast, the solid VFLS sleeve guarantees accurate centering of each screw within the cis cortex, facilitating consistent and reproducible progressive dynamization. This feature ensures consistent application of the same biomechanical stimulus across all patients and can contribute to lowering complication rates: for example, in medial opening wedge high tibial osteotomies.

Taken together, the biomechanical, preclinical, and this first observational study in humans suggest that the variable locking screw technique is a safe technique and has the potential to promote bone regeneration in osteotomy and fracture treatment. This potential is particularly important in the treatment of osteoporotic fractures and in the prevention of complications such as non-union. The use of more effective devices in elective surgical procedures can provide significant benefits, especially when these procedures are performed in ambulatory surgical centers [49], in particular today. In fact, there is growing evidence that patients who undergo HTO may still be suitable candidates for medial unicompartmental knee replacement in the future [50], challenging the traditional view of HTO as an obstacle to knee arthroplasty. Moreover, the flexibility of converting from a medial UKR to a total knee replacement (TKR), if necessary, provides an added advantage, offering surgeons more options for optimal long-term management of knee joint pathology [51,52,53,54,55].

This first observational study presents several limitations that must be considered when interpreting its findings. One key limitation is the relatively small sample size, with a limited number of patients included, which reduces the statistical power and the broader applicability of the results. Additionally, the correction angle applied in this cohort is relatively low in comparison to those reported in other studies with which we are qualitatively comparing our results. This discrepancy in correction angles may impact the generalizability of our findings, as different degrees of correction could yield varying outcomes in terms of functional improvement and biomechanical conditions. Furthermore, it is important to acknowledge that the surgeries in this study were performed exclusively by a single, experienced surgeon, which introduces a potential bias and limits the external validity of the results. The expertise and techniques of one surgeon, while high-quality, may not fully represent the outcomes achievable in a broader, more diverse clinical setting with multiple practitioners. Therefore, the generalizability of this study’s conclusions to other clinical environments and practitioners should be approached with caution.

## 5. Conclusions

This initial observational clinical study provides evidence that Variable Fixation Locking Screws can be safely and effectively employed in the treatment of medial opening wedge high tibial osteotomies. The results from this study are encouraging, as no complications, adverse events, or necessity for implant removal were reported. At the six-month follow-up, 70% of patients demonstrated both radiographic and clinical evidence of healing, while 100% achieved complete functional recovery without experiencing issues such as limb length discrepancy, joint instability, pain, or stiffness. These outcomes suggest that the use of VFLSs effectively support bone consolidation and contribute to favorable postoperative results in this patient population.

As surgeons, it is our responsibility to advocate for the continuous advancement of available technologies in order to meet the evolving needs of our patients. We must also challenge the mindset that “no further improvement is possible”. The history of surgery has consistently shown that even small changes in surgical techniques or the design of implants can result in significant clinical benefits. Developing new devices and techniques that help mitigate complications commonly associated with traditional fixation methods, such as delayed union or non-union, is critical to addressing the needs of an aging yet active population.

Although the preliminary findings of this study are encouraging, further research is required to thoroughly assess the clinical efficacy and substantiate these results. Future investigations should involve larger patient cohorts, multicenter trials, and the inclusion of control groups to generate comprehensive, reliable evidence regarding the effectiveness of Variable Fixation Locking Screws in promoting fracture healing and minimizing complications. These studies are critical for confirming the potential benefits of this innovative fixation technology and for ensuring its broader implementation and adoption in clinical practice.

## Data Availability

The data presented in this study are available on request from the corresponding author.

## Supplementary Materials

The following supporting information can be downloaded at https://www.mdpi.com/article/10.3390/jcm13247707/s1: Table S1: Inclusion and exclusion criteria.
