# Supplementary material for: Positive Results Using Variable Fixation in Medial Opening Wedge High Tibial Osteotomies in Patients with Unilateral Knee Osteoarthritis: An Observational Clinical Investigation
Source: J Clin Med. 2024 Dec 17;13(24):7707. doi: 10.3390/jcm13247707 (PMC11678760; doi:10.3390/jcm13247707)
Supplement: Supplementary file 1 [file jcm-13-07707-s001.zip › jcm-3332419-supplementary.pdf]

Supplementary Materials

Table S1. Inclusion and exclusion criteria

Inclusion criteria	• Corrective osteotomies where the principal investigators see the possibility to use 5.0 mm screws in cortical bone.<br>• Patient aged 18 years and older at the time of the trauma.<br>• Ability to walk independently prior to injury.<br>• Eligible for treatment with Open Reduction and Internal Fixation (ORIF).<br>• Willing and able to comply with postoperative protocol and return for follow-up.
Exclusion criteria	• Any not-medically managed severe systemic disease (e.g., diabetes, hyperthyroidism, renal insufficiency).<br>• Re-fracture on the same bone segment.<br>• Chronic inflammatory diseases (e.g., celiac disease, vasculitis, lupus, chronic obstructive pulmonary disease (COPD), irritable bowel disease, psoriasis).<br>• Pregnancy, women planning to conceive within the study period, or breastfeeding.<br>• Active malignancy.<br>• Admission of immune response-modulating drugs.<br>• HIV or chronic hepatitis B/C.<br>• Osteitis or osteomyelitis.<br>• Allergic reaction to Ti alloy, lactic, or glycolic acid.<br>• Pre-existing malunion or non-union of the ipsilateral lower extremity.<br>• Segmental bone defect requiring bone grafting.<br>• Pathologic fracture due to a disease other than osteoporosis (e.g., tumor, metastasis).<br>• Recent (6 months) history of substance abuse (i.e., recreational drugs, alcohol) that would preclude reliable assessment.<br>• Prisoner.<br>• Participation in any other medical device or medicinal product study within the previous month that could influence the results of the present investigation.<br>• Lack of bone substance or poor bone quality which, in the surgeon’s judgment, makes locked plate fixation impossible.<br>• Addition of a bone graft, bone graft substitute, or bone morphogenetic proteins (BMPs).
